# Long-Term Persistence of *bla*_CTX-M-15_ in Soil and Lettuce after Introducing Extended-Spectrum β-Lactamase (ESBL)-Producing *Escherichia coli* via Manure or Water

**DOI:** 10.3390/microorganisms8111646

**Published:** 2020-10-23

**Authors:** Maria-Theresia Gekenidis, Serena Rigotti, Jörg Hummerjohann, Fiona Walsh, David Drissner

**Affiliations:** 1 Microbiological Food Safety, Agroscope, 8820 Wädenswil, Switzerland; serena.rigotti@alumni.ethz.ch; 2 Microbiological Food Safety, Agroscope, 3003 Liebefeld, Switzerland; joerg.hummerjohann@agroscope.admin.ch; 3 Department of Biology, Maynooth University, W23 F2H6 Maynooth, Co. Kildare, Ireland; Fiona.Walsh@mu.ie; 4 Department of Life Sciences, Albstadt-Sigmaringen University, 72488 Sigmaringen, Germany; drissner@hs-albsig.de

**Keywords:** antibiotic resistance, irrigation water, manure, lettuce, ESBL-producing *E. coli*, *bla*_CTX-M-15_, persistence

## Abstract

The number of environmental antibiotic-resistant bacteria (ARB) has increased dramatically since the start of antibiotic mass production for broad bacterial infection treatment in 1944. Nowadays, ARB and their resistance-determining genes (ARGs) are readily detected in all environments, including the human food chain. A highly relevant food group in this context is fresh produce, frequent raw consumption of which facilitates direct transfer of ARB and ARGs to the consumer. Here, we investigate the persistence of an extended-spectrum β-lactamase (ESBL)-producing *Escherichia coli* (*E. coli*) pEK499 and its clinically most important ARG (*bla*_CTX-M-15_), after introduction via irrigation water or manure into a lettuce-growing system. Culturable ESBL-producing *E. coli* persisted longest in soil and when introduced via manure (until 9 weeks after introduction), while being undetectable on lettuce beyond day 7. In contrast, qPCR detection of *bla*_CTX-M-15_ was much more frequent: introduction via water significantly increased *bla*_CTX-M-15_ on lettuce until week 4, as opposed to manure, which affected the soil in the long-term (9 weeks) while leading to *bla*_CTX-M-15_ detection on lettuce until day 7 only. Our findings demonstrate long-term persistence of undesired ARB and ARG after their introduction via both irrigation and amendment. Such an understanding of the persistence kinetics of an ESBL-producing *E. coli* and plasmid-encoded *bla*_CTX-M-15_ aids the determination of critical actions in order to mitigate their transfer to the consumer.

## 1. Introduction

The conditions under which fresh produce is grown vary largely worldwide. Conditions such as the produce’s growing environment (open field, greenhouse, hors-sol), type of fertilizer (e.g., inorganic, livestock manure, or compost), or quality of irrigation water (from drinking water to reclaimed wastewater) are chosen depending on the kind of produce, farming practice (conventional vs. organic), available resources, and the applied legal framework. Whichever the combination, fresh produce is exposed to a variety of potential contamination sources. Among the main sources are irrigation water, soil, and applied fertilizer [1].

In terms of contaminants, much attention has been payed to foodborne pathogens, which are transported by the various contamination sources to the produce and can thereafter cause outbreaks, which are well-documented and monitored [2,3]. Apart from pathogens, however, antibiotic-resistant bacteria (ARB) are just as probable to contaminate fresh produce and survive harvest and processing, thereby ending up on the consumer’s plate. These cases are by far not as well-documented, as they do not result in direct harm of the consumer. However, reports of produce-borne ARB of clinical importance such as extended-spectrum β-lactamase (ESBL)-producing or colistin-resistant *Enterobacteriaceae* or vancomycin-resistant enterococci [4,5,6,7], as well as the documented ability of foodborne bacteria to survive upon ingestion to the human gastro-intestinal tract and interact with the gut-resident microbiota [8] underline that ARB are not negligible food contaminants. Especially when carrying horizontally transferable resistance-determining genes (ARGs), ARB can contribute to the spread and establishment of these genes in the gut [9]. This so-called gut resistome has the potential to transfer ARGs to opportunistic pathogens inhabiting the gut or pathogens entering at a later point in time [10], thereby impeding effective disease treatment. This should not be underestimated, since a significant percentage of healthy individuals worldwide are colonized by ESBL-producing *Enterobacteriaceae* [11].

Irrigation water is an important contamination source of produce with ARB and ARGs, especially when coming into direct contact with the consumed plant parts. The quality of water used for irrigation varies immensely, from potable tap water over surface waters to reclaimed wastewater [12]. Wastewater treatment plants often release treated wastewater into nearby streams, thereby contributing to an increase in ARB and their resistance determinants in surface waters [13,14,15]. Despite this large variance in quality and acknowledged presence of ARB, ARGs, and other contaminants [12,16], irrigation water is not monitored routinely to minimize contamination of the irrigated produce. It is therefore important to understand the persistence kinetics of ARB and ARGs on the produce and in the farming system.

Due to the high use of antibiotics in animals, manure can disseminate residues of active antibiotic as well as ARB [17,18]. Produce harvested from manure-amended fields can therefore contain an increased burden of ARB and ARGs of either enteric or environmental origin [19]. Storage or other practices such as composting of manure prior to application are therefore recommended [20,21,22]. Additionally, wait times between manure application and harvest are practiced in certain regions, such as the 90 to 120 days recommended by the US National Organic Program, depending on whether the edible parts come into direct contact with the manure-amended soil or not [23]. For leafy greens including salads, the worldwide standard for good agricultural practices (Global G.A.P.) prescribes incorporation of raw manure into the soil at least 60 days prior to harvest and prohibits its application after planting (control point FV 4.2.1, [24]). In Switzerland as well, slurry must be incorporated into the soil before sowing or planting. In vegetables with a long cropping period, slurry is permitted, but leaf contact must be avoided, and the last application should take place 6 weeks before harvest [25].

Upon introduction into an agricultural system, ARB can get into direct contact with edible plant parts, for example through overhead irrigation [26]. Additionally, ARB can become subject to internalization—through either natural apertures, such as leaf stomata or lateral root junctions, or through wounds [27]. Internalization of *E. coli* after introduction via manure into the soil has been demonstrated [28]. Once internalized into the roots, bacteria can be transferred systemically to reach the aerial plant organs. This must be taken into consideration, since internalized bacteria are more likely to survive post-harvest sanitation of the food product [27].

Clinically significant ARB have been classified into concerning, serious, and urgent threats [29]. The arsenal of effective antibiotics to treat infections with ARB of the serious and urgent threat groups is very limited. Among the serious threats, the U.S. Center for Disease Control and Prevention lists ESBL-producing *Enterobacteriaceae*, including ESBL-producing *E. coli* [29]. ESBLs are enzymes capable of hydrolyzing and thereby inactivating penicillins as well as first-, second-, and third-generation cephalosporins, and aztreonam. According to the latest surveillance of antimicrobial resistance by the European Centre for Disease Prevention and Control (ECDC), 15% of all *E. coli* isolates reported to the European Antimicrobial Resistance Surveillance Network (EARS-Net) for 2018 were resistant to third-generation cephalosporins [30]. Genes encoding ESBLs have been detected on transmissible plasmids in environments ranging—apart from the clinics—from wild, domestic, or companion animals over various foods (including fresh produce) to surface waters and soil [5,31,32,33,34,35]. Until recently, over 500 β-lactamases had been reported, the spread of which is often linked to plasmid-mediated ESBLs, and more specifically ESBLs of the CTX-M family [36]. Of these, the two variants *bla*_CTX-M-15_ and *bla*_CTX-M-14_ are the most common ones.

In the present study, we investigated survival and persistence of ESBL-producing *E. coli* pEK499 and its plasmid-borne *bla*_CTX-M-15_ gene [37] in soil and lettuce grown under applied conditions. The multidrug-resistance (MDR) plasmid pEK499 is a 117.5 kbp large and low-copy plasmid (1–2 copies per cell [38]). It comprises ten ARGs as well as five plasmid maintenance systems [37], which ensure very stable plasmid inheritance. *Escherichia coli* pEK499 was introduced through either irrigation water (low- and high-level contamination) or manure application, while in the latter the recommended wait time between application and harvest was respected. To track the development of culturable ESBL-producing *E. coli* as well as *bla*_CTX-M-15_ through the early food chain, that is, from their introduction until harvest, we analyzed water, manure, leaf, and soil material for the presence of culturable *E. coli* and quantified *bla*_CTX-M-15_ by quantitative PCR (qPCR).

## 2. Materials and Methods

If not specified otherwise, material was purchased from Sigma-Aldrich (St. Louis, MI, USA).

### 2.1. Resistance Testing and Preparation of Inoculation Cultures

The ESBL-producing *E. coli* carrying the MDR plasmid pEK499 [37] was used in all experiments. To determine appropriate antibiotics for selecting *E. coli* pEK499 from the non-sterile lettuce-in-pot system, the strain was screened in triplicate against 32 clinically relevant antibiotics by disk diffusion, as described previously [26]. Resistance was determined using the clinical breakpoint tables of the European Committee on Antimicrobial Susceptibility Testing (version 10.0; EUCAST). Where breakpoints had been removed, the previous cutoffs were used (temocillin, cephalothin, kanamycin, nalidixic acid, sulfonamide, minocycline, and colistin).

For inoculation of water or manure with *E. coli* pEK499, frozen bacterial culture was streaked on CHROMagar *E. coli* plates (CHROMagar^TM^, Paris, France) containing ampicillin (AMP, 32 mg/L) and tetracycline (TET, 8 mg/L). After incubation at 37 °C for 24 h, a liquid culture was grown from a single colony in LB broth with 32 mg/L AMP (agitated at 37 °C, overnight). An appropriate volume of liquid culture was centrifuged and washed twice with PBS (8 g NaCl, 0.2 g KCl, 1.15 g Na_2_HPO_4_, and 0.2 g KH_2_PO_4_ in 1 L distilled water; pH 7.3) to remove medium and antibiotic residues. After resuspension in PBS, optical density at 600 nm (OD_600_) was determined and—based on a previously established correlation between OD_600_ and colony-forming units of *E. coli* pEK499—an appropriate amount of OD_600_-adjusted bacterial suspension was added to tap water or manure.

### 2.2. Lettuce-in-Pot: Growth Conditions and Inoculation

#### 2.2.1. Setup

Field soil was steam-treated (approx. 4 h, 90 °C) to inactivate weed and microorganisms, allowed to dry, sieved to remove stones and large particles (5 mm mesh size), and aliquoted into plastic bags. Plastic pots and pot-coasters were prepared in a biosafety-level 2 greenhouse (G-II) by adding two glass microfiber filters (9 cm diameter, 1.6 µm pores; Lab Logistics Group GmbH, Meckenheim, Germany) into each pot to avoid soil leakage and by numbering the pots for randomized sampling of soil and lettuce over the complete growth period: the pots were first randomly assigned to either treatment or control (with or without *E. coli* pEK499 added to water or manure, respectively) and then randomly assigned to sampling time points. To obtain lettuce seedlings, *Lactuca sativa* (Salanova^®^ Barlach) seeds were seeded into trays containing seedling soil and grown in a regular greenhouse. The conditions in the G-II cabin were a software-controlled day–night cycle for optimal lettuce growth: 16 h light with 20 °C, 8 h darkness with 15 °C, and 50% relative humidity.

#### 2.2.2. Water-Inoculation Experiments

For the water-experiments, the pots were filled with the pre-aliquoted soil (approx. 500 g), and three-week-old lettuce seedlings were immediately planted (one seedling per pot). Plants were watered regularly according to their needs using tap water while paying attention to not disturb the leaves. After one week, a one-time contamination was simulated by irrigating a random 50% of the lettuces overhead with (A) 100 mL of tap water per plant, containing 10^4^ CFU/mL *E. coli* pEK499 (low-level homogeneous contamination) or (B) 10 mL of tap water per plant, containing 10^6^ CFU/mL (high-level, splash-like contamination). The bacterial loads chosen for conditions (A) or (B) correspond to realistic levels of *E. coli* in surface waters or water splashing from soil onto lettuce, respectively [39,40]. The control plants were irrigated as usual, using tap water.

#### 2.2.3. Manure-Inoculation Experiment

Swine manure (obtained from Agroscope, Posieux) was sieved to remove straw and was stored in plastic containers at room temperature until use. Respecting the guidelines defining maximum levels of fertilizer-introduced nitrogen for field-grown lettuce, 80 mL of swine manure (with 1.36 g of nitrogen per kg) were added to soil pre-aliquoted in plastic bags (approx. 470 g of soil per bag). For half the bags, manure containing 10^8^ CFU/mL *E. coli* pEK499 was used (treatment group), while native manure was added to the other bags (control group). Soil and manure were mixed thoroughly inside the bags and then transported into the G-II cabin, where the mixture was transferred to the randomly assigned treatment or control pots, respectively. During a quarantine period of 6 weeks—in order to respect the recommended 60 days between manure application and salad harvest—pot soil was wetted with tap water regularly to avoid over-drying. After the quarantine, three-week-old seedlings were planted (one per pot) and watered regularly using tap water.

### 2.3. Sampling and Culture-Based Approach

#### 2.3.1. Starting Materials and Sampling Scheme

The tap water used for regular irrigation of plants was analyzed at the beginning and end of each experiment, to ensure the absence of generic and ABR-*E. coli*. Biological replicates of the tap water were drawn on three consecutive days. Each time, three liters of water were pre-filtered using nitrocellulose (NC) filters (5 μm pore size; Merck Millipore, Burlington, MA, USA). Thereof, technical replicates at 100 mL were filtered through NC filters (0.22 μm pore size; Merck Millipore) for direct incubation in duplicate on CHROMagar *E. coli* either without or with AMP32/TET8 (designated CrA(-) and CrA(+) in the following; 37 °C for 24 h). Additionally, two liters of pre-filtered water per biological replicate were filtered using polycarbonate (PC) filters (0.22 μm pore size; Merck Millipore) for subsequent DNA extraction.

On the day of planting, random samples of pot soil, seedling soil, and seedling leaves were drawn for detection of total and AMP/TET-resistant *E. coli* as well as *bla*_CTX-M-15_, potentially introduced into the system by these starting materials. These soil and leaf samples were processed as described below for the corresponding sample types. Repeated samplings were conducted from inoculation with *E. coli* pEK499 to final lettuce harvest, by collecting lettuce leaves and soil from the randomly assigned pots per time point (three for each control and treatment group).

Samplings were conducted as follows: In the low- and high-level contamination water experiments (W-lo and W-hi), triplicate soil and lettuce samples were collected on the day of inoculation (dpi0) as well as on day 1 post inoculation (dpi1), dpi3, and the weeks one to four, post inoculation (dpi7 to dpi28). In the manure experiment, duplicate soil samples were taken during the quarantine period (that is, before lettuce planting), on the day of manure inoculation (pre-dpi0) as well as pre-dpi1 and pre-dpi3, and weekly thereafter (pre-wpi1 to pre-wpi5). When lettuces were planted, triplicate soil and lettuce samples were collected on the day of planting (dpi0), dpi3, and dpi7 to dpi21.

#### 2.3.2. Lettuce Leave Sampling

Per replicate, 20 g of leaves were collected in 1 L sterile plastic bottles using tweezers and scissors, which were sterilized in 70% ethanol at the start and between replicates. In the laboratory, 180 mL of PBS were added to each bottle, and the leaves were gently pushed into the liquid using a Drigalski spatula until they were all covered by the buffer. The six bottles were sonicated for 7 min to dislodge the bacterial cells from the leaves, while turning all bottles at half-time to ensure symmetric sonication.

For the manure experiment, leaves were equally collected and diluted with PBS. However, to ensure extraction of potentially internalized bacteria—since *E. coli* pEK499 had been mixed into the soil before planting, rather than being introduced by overhead irrigation—stomacher TEMPO^®^ bags with incorporated lateral filters (BioMérieux, Marcy-l’Étoile, France) were used for leaf collection and the samples were processed for 3 min in a stomacher (Smasher; AES Chemunex, Combourg, France) instead of sonication.

The thus generated leaf wash (water-inoculation) or leaf homogenate (manure-inoculation) was pre-filtered through NC filters (5 µm pores), and each eluate was collected in a separate sterile flask. Thereof, 100 mL were filtered through PC filters (0.22 μm pores), which were stored at −80 °C for subsequent DNA extraction. Of the remaining leaf wash/homogenate, 2 × 10 mL were filtered through NC filters (0.22 µm pores), which were then incubated on CrA(-) and CrA(+) (37 °C for 24 h), and 100 μL of undiluted and 10-fold diluted pre-filtered leaf wash/homogenate were plated in duplicates on CrA(-) and CrA(+). Finally, colonies displaying the *E. coli*-typical blue coloration as well as white secondary colonies were enumerated.

#### 2.3.3. Soil Sampling

Starting from the planting day (water experiments) or the day of manure inoculation (manure experiment), four soil cores (1.8 cm diameter, 10 cm depth) were sampled per pot and collected in a sterile plastic bag. Each sample was then homogenized by sieving (1 mm mesh size), and three subsamples were taken. First, 3 × 250 mg were stored at −80 °C for later DNA extraction. Second, 10 g of soil were transferred into sterile glass and diluted with 90 mL of PBS. After thoroughly shaking the suspension (200 rotations per min, 1 min), large particles were allowed to settle for 5 min. Thereafter, 30 mL of the supernatant were transferred into sterile plastic tubes, and 10-fold dilutions were produced in PBS. One mL and 100 μL of the supernatant as well as appropriate dilutions were plated onto CrA(-) and CrA(+) in duplicates and incubated (37 °C for 24 h) for subsequent enumeration. Finally, soil dry weight was determined with a moisture analyzer (model HC103; Mettler Toledo, Columbus, IN, USA) at 160 °C, using 10 g of wet soil. Dry weight was registered when the weight stabilized at mg-scale for at least 50 s.

In addition to direct culturing, enrichments of *E. coli* from leaf homogenates as well as soil suspensions were performed in EE broth Mossel (Beckton Dickinson, Franklin Lakes, NJ, USA). After incubating 1 mL of leaf wash/homogenate or soil suspension, respectively, in 9 mL of EE broth (37 °C for 24 h), 10 μL of the enrichment were streaked onto CrA(+), and plates were examined for colonies after 24 h at 37 °C.

### 2.4. ARG Detection in Re-Isolated E. coli pEK499 and Secondary MDR Colonies

Bacterial single colonies showing *E. coli*-typical blue coloration as well as secondary white colonies were picked from CrA(+) plates of soil and leaf washes/homogenates, re-grown in LB broth with AMP32/TET8, and stored with 15% glycerol at −80 °C. Genomic DNA was extracted from selected blue- and white-colored colonies (up to three per replicate and colony type) using the GenElute^TM^ Bacterial Genomic DNA kit.

A triplex PCR for detection of three ARGs (*aadA5*, *bla*_CTX-M-15_, and *tetA*) carried by plasmid pEK499 was established using custom-synthesized primers (Microsynth, Balgach, Switzerland; Table A1 and Table A2) and Phusion Hot Start II High-Fidelity Master Mix (Thermo Fisher Scientific, Waltham, MA, USA). *Escherichia coli* pEK499 was used as the positive control, while *E. coli* ATCC 25922 and distilled water served as negative controls. Bands were visualized on a 1 × TAE gel (0.8% agarose, 120 min, 85 V) with GelRed^®^ (Biotium, Fremont, CA, USA). White colonies yielding all three ARG bands were identified at species level using Matrix-Assisted Laser Desorption Ionization (MALDI) biotyping as described previously [41].

### 2.5. Molecular Detection of bla_CTX-M-15_ in Plant and Soil

For soil, DNA extracts were generated from 250 mg of soil for each sampled replicate, using the DNeasy PowerSoil Kit (Qiagen, Venlo, Netherlands) according to instructions. For lettuce, the PC filters containing DNA of 100 mL pre-filtered leaf wash/homogenate were used with the DNeasy PowerWater Kit (Qiagen) to generate DNA extracts of all lettuce samples according to instructions. Quantity and quality of the extracted DNA were measured on a NanoDrop^TM^ One Spectrophotometer (Thermo Fisher Scientific). Finally, all extracts were tested for amplification of the bacterial 16S rRNA gene (universal primers 27F and 1492R, Table A1) using DreamTaq^TM^ PCR Master Mix 2X (Thermo Fisher Scientific), to ensure absence of PCR inhibitors before starting qPCR.

Quantitative PCR for detection of *bla*_CTX-M-15_ was conducted on a ViiA^TM^ 7 System (Applied Biosystems, Waltham, MA, USA) using TaqMan™ Multiplex Master Mix (Thermo Fisher Scientific) in MicroAmp™ Fast Optical 96-Well Reaction Plates (Applied Biosystems) covered with MicroAmp^TM^ Optical Adhesive Film (Applied Biosystems). Primers (5′- ATC ACG CGG ATC GCC CGG AAT-3′ and 5′-ATG TGC AGC ACC AGT AAA GTG ATG GC-3′ [42]) and a probe (5’-CCC GAC AGC TGG GAG ACG AAA CGT-3’) with reporter/quencher FAM/BHQ-1 were ordered at Microsynth. The run method consisted of a hold stage (10 min at 95 °C) and a PCR stage with 45 cycles (8 sec at 95 °C, 60 sec at 58 °C). Twenty microliter reactions including 5 μL of template DNA were prepared, containing 0.5 μM final concentrations of each primer as well as the probe. For absolute quantification of *bla*_CTX-M-15_, a GenElute^TM^ DNA extract from a known number of *E. coli* pEK499 (~ 10^8^ CFU) was used. A standard curve was generated by measuring ten-fold dilutions of the extract in duplicate on all reaction plates, and *bla*_CTX-M-15_ copies were calculated assuming one plasmid copy per bacterial cell. Experiments were set up, and raw data were analyzed using QuantStudio^TM^ Real-Time PCR Software (version 1.3; Thermo Fisher Scientific).

### 2.6. Statistics

To compare control to treated system (either lettuce or soil) per time point, unpaired two-sample t-tests were conducted on log-transformed data using GraphPad Prism 8.4.3 (GraphPad Software, San Diego, CA, USA). The Holm–Sidak method was applied to correct for multiple testing. Significant differences (*p* < 0.05) were marked with an asterisk.

## 3. Results

### 3.1. Survival of E. coli pEK499 in Lettuce-in-Pot System

#### 3.1.1. Pre-Inoculation

**Figure 1:**
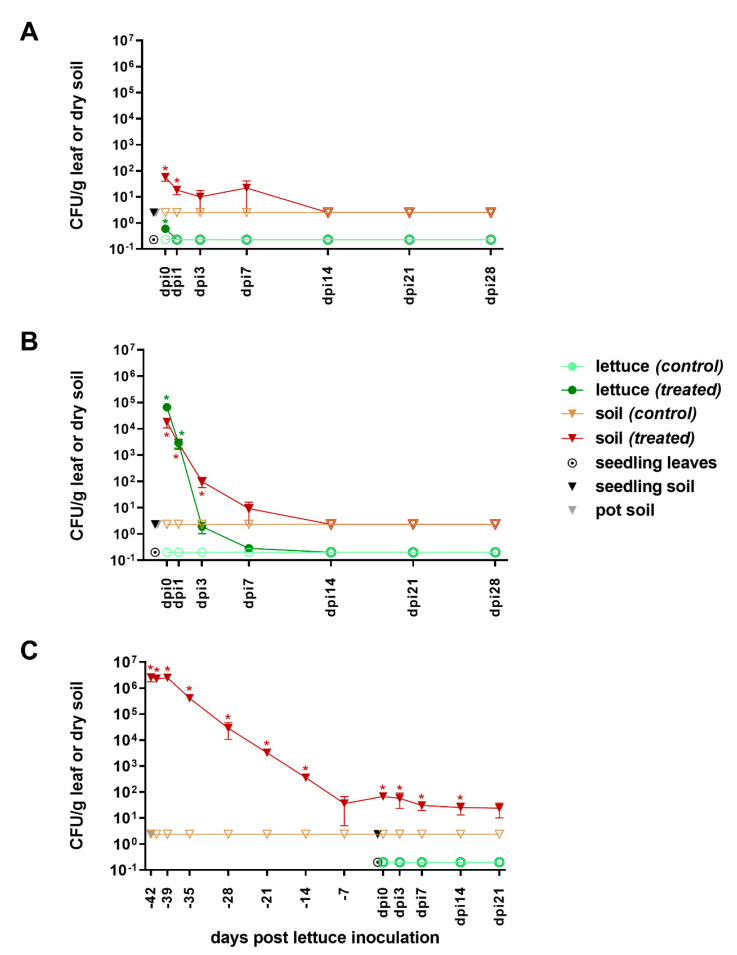
Survival of *E. coli* pEK499 in soil (-▼-) and lettuce (-●-) from its introduction until harvest. Low- and high-level contamination experiments via water (**A** and **B**, respectively) and via manure (**C**) are shown. Empty symbols indicate values below the respective limit of detection. Error bars indicate standard error of the mean (SEM, *n* = 3). Significant differences between treatment and control (*p* < 0.05) are marked with an asterisk. A detailed view of panel A is provided in Figure A1.

The ESBL-producing *E. coli* pEK499 selected for this study carries ten ARGs on a 117.5 kbp plasmid (*bla*_TEM-1_, *bla*_OXA-1_, *bla*_CTX-M-15_, *aac6′-Ib-cr*, *mph*(A), *catB4*, *tetA*, *aadA5*, *dfrA7*, and *sul1*) conferring resistance to eight antibiotic classes [37]. Blue colonies growing on CrA(+) containing antibiotics from two of these classes (β-lactams and tetracyclines) were counted as *E. coli* pEK499. This procedure is valid since samples of the starting materials—that is, water, manure, pot soil, seedling soil, and seedling leaves—never resulted in growth of blue colonies on CrA(+) (seedling leaves/soil and pot soil shown in Figure 1), demonstrating that no AMP/TET-resistant *E. coli* were introduced into the system prior to inoculation with *E. coli* pEK499. The culture data presented below therefore represent *E. coli* pEK499.

Moreover, the starting materials neither contained susceptible *E. coli* as shown by culturing on CrA(-), except for seedling soil and manure. Seedling soil on one hand contained susceptible *E. coli* in low amounts (W-lo: 250 ± 10 CFU/g dry soil; W-hi: 11 ± 8 CFU/g dry soil; M: 62 ± 13 CFU/g dry soil) and made up a small portion of the whole system, wherefore susceptible *E. coli* were hardly ever detected in the pEK499-free control system after transferring seedlings into pots (only in 2 of 72 samples collected from all three experiments). Manure, on the other hand, also contained a negligible number of susceptible *E. coli*: only 1 of 30 control soil samples—that is, soil mixed with pEK499-free manure—was positive with 5 CFU/g dry soil, and *E. coli* counts of pEK499-inoculated manure did not differ significantly between CrA(-) and CrA(+) (both ~ 1.1 × 10^8^ CFU/mL). Due to these rare and low counts of susceptible *E. coli*, the possibility of *E. coli* pEK499 counts including transconjugants can be neglected.

#### 3.1.2. From Inoculation to Harvest

**Water (low-level).** When irrigated with a low level of ESBL-producing *E. coli* (10^4^ CFU/mL, 100 mL per plant) mimicking overhead irrigation with contaminated surface water, we observed that most *E. coli* ended up in the soil (Figure 1A). On the day of inoculation, 5.8 × 10^1^ CFU/g dry soil were detected in the treated system, whereas treated lettuce had two orders of magnitude less of AR *E. coli* (6.0 × 10^−1^ CFU/g). Already from the first day after inoculation (dpi1) and until the end of the experiment (dpi28), AR *E. coli* were below the limit of detection on the lettuce leaves (< 2.3 × 10^−1^ CFU/g). In contrast, AR *E. coli* were culturable from treated soil until one week after inoculation (dpi7) at around 2.0 × 10^1^ CFU/g and were undetectable (< 2.5 × 10^0^ CFU/g) in the three weeks thereafter (from dpi14 to dpi28). In the control system, no AR *E. coli* was detected in either soil or lettuce at any time as expected.

**Water (high-level).** When mimicking a splashing event by irrigating each plant with 10 mL of high-level ESBL-producing *E. coli* (10^6^ CFU/mL), AR *E. coli* were detectable on lettuce much longer compared to the low-level experiment, that is, until dpi7 (Figure 1B). About 6.7 × 10^4^ CFU/g of leaf were isolated from freshly inoculated plants (dpi0). However, the number of AR *E. coli* on plant leaves decreased exponentially, reaching about 2.8 × 10^−1^ CFU/g on dpi7 and further decreasing below detection (< 2.0 × 10^−1^ CFU/g) in the subsequent samplings (dpi14, 21, and 28). In soil, AR *E. coli* also decreased—albeit with a slower rate than on the leaves—from 1.8 × 10^4^ CFU/g at dpi0 to 9.2 × 10^0^ CFU/g at dpi7 (Figure 1B). Thereafter (from dpi14 to dpi28), AR *E. coli* were not detectable in soil anymore. As expected, no AR-*E.coli* were detected at any time in soil and on leaves of the control system.

**Manure.** Manure containing *E. coli* pEK499 was introduced into pot soil 6 weeks prior to planting of lettuces, resulting in 2.6 × 10^6^ CFU of AR *E. coli* per gram dry soil on the day of inoculation (day -42, Figure 1C). After staying around 2.5 × 10^6^ CFU/g for the first three days, a log-linear decrease was observed during the complete quarantine period, until bacterial numbers reached 3.6 × 10^1^ CFU/g dry soil one week prior to lettuce planting. At that time, AR *E. coli* numbers stabilized and remained detectable at around 4 × 10^1^ CFU/g until the end of the experiment. In contrast to treatment soil, soil mixed with non-inoculated manure (control) contained no detectable AR *E. coli* (< 2.4 × 10^0^ CFU/g) throughout the experiment. As anticipated, no AR *E. coli* were detectable in control lettuce leaves (< 2.0 × 10^−1^ CFU/g). Interestingly, no AR *E. coli* were ever detected in leaves of treated lettuces, despite the continuous presence of AR *E. coli* in the soil they were grown in.

Overall, when introduced via water, a rapid decrease in *E. coli* pEK499 on lettuce was observed, while in soil, the numbers decreased more slowly (Figure 1A,B). Nevertheless, no significant differences between treated and untreated systems were detected beyond dpi3. Manure application, on the other hand, resulted—after a log-linear decrease during the first 35 days—in a persistent low load of *E. coli* pEK499 in soil (Figure 1C). Of note, enrichment of *E. coli* from soil or leaf washes/homogenates by use of EE broth did not yield any additional information, since it was negative whenever direct plating was negative.

### 3.2. Detection of bla_CTX-M-15_ in the Lettuce-in-Pot System

#### 3.2.1. Pre-Inoculation

**Figure 2:**
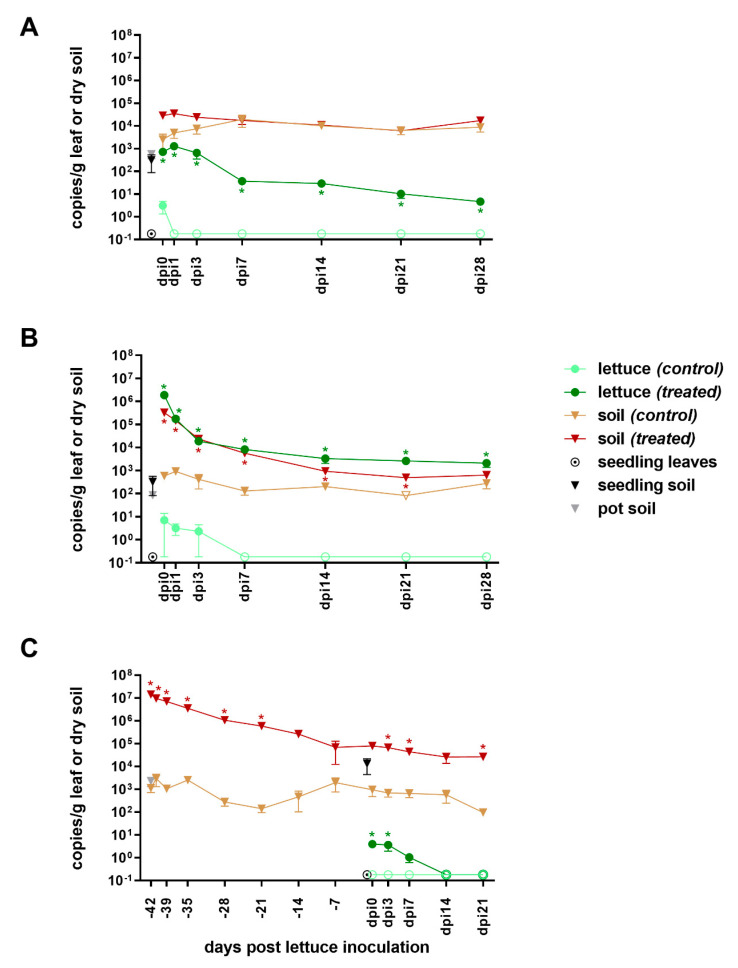
Detection of *bla*_CTX-M-15_ in soil (-▼-) and lettuce (-●-) from introduction of extended-spectrum β-lactamase (ESBL)-producing *E. coli* pEK499 until harvest. Low- and high-level contamination via water (**A** and **B**, respectively) and via manure (**C**) are shown. Empty symbols indicate values below the respective limit of detection. Error bars indicate standard error of the mean (SEM, *n* = 3). Significant differences between treatment and control (*p* < 0.05) are marked with an asterisk.

In parallel to quantifying culturable ESBL-producing *E. coli* from soil and lettuce leaves, we performed qPCR on DNA extracted from the same environmental samples to quantify *bla*_CTX-M-15_, the ARG conferring the ESBL-phenotype to *E. coli* pEK499. We expected to detect a background load of *bla*_CTX-M-15_ in non-inoculated soil, as it is an ARG often detected in the environment. In fact, we detected *bla*_CTX-M-15_ in the soils used to set up the system—that is, seedling and pot soil—over all experiments between 8.3 × 10^1^ and 3.9 × 10^3^ copies/g dry soil (Figure 2). Consequently, the applied steam treatment (approx. 4h, 90 °C) was not able to result in complete degradation of this ARG in the soil. No *bla*_CTX-M-15_ was detected, however, in the repeatedly sampled irrigation water or on seedling leaves sampled at the start of each experiment (Figure 2).

#### 3.2.2. From Inoculation to Harvest

**Water (low-level).** In the control system, *bla*_CTX-M-15_ was detected at 2.5 × 10^3^ copies/g soil on the day of inoculation, that is, increased by almost one order of magnitude as compared to the starting materials seedling and pot soil, which were around 4.4 × 10^2^ copies/g (Figure 2A). On the following samplings, *bla*_CTX-M-15_ further increased gradually, until it reached 2.0 × 10^4^ copies/g on dpi7. Thereafter, *bla*_CTX-M-15_ copy numbers slightly decreased again reaching 6.4 × 10^3^ and 8.8 × 10^3^ copies/g in the third and fourth week after inoculation, respectively. Lettuce leaves harvested from the control system also had detectable levels of *bla*_CTX-M-15_ on the day of inoculation (3.1 × 10^0^ copies/g, Figure 2A). However, *bla*_CTX-M-15_ was not detectable anymore in all subsequent samplings of control lettuce leaves (< 1.8 × 10^−^^1^ copies/g). On the other hand, *bla*_CTX-M-15_ was constantly detected in soil and on lettuce leaves of the treatment group throughout the experiment. In soil, 2.9 × 10^4^ copies/g were measured on dpi0, only slightly decreased to 6.2 × 10^3^ copies/g until dpi21, and increased again until dpi28 (1.7 × 10^4^ copies/g). Overall, the loads were very similar to the soil of the control system, with the exception of the first three days after inoculation. On the contrary, leaves from treated lettuces had significantly higher *bla*_CTX-M-15_ copy numbers than control lettuce leaves for the whole sampling period. Starting at 7.3 × 10^2^ copies/g on dpi0, *bla*_CTX-M-15_ copy number increased to 1.3 × 10^3^ copies/g, remained high until the third day, and then slowly decreased until dpi28, where it was still clearly detectable with 4.6 × 10^0^ copies/g.

**Water (high-level).** After inoculating half of the plants with a small volume of water containing a high load of *E. coli* pEK499, *bla*_CTX-M-15_ was more abundant on the leaves than in soil, amounting to 1.9 × 10^6^ copies/g vs. 3.3 × 10^5^ copies/g dry soil (Figure 2B). Thereafter, the time trend on leaves and in soil was comparable, consisting of an initial steep decrease during the first three days to about 2.1 × 10^4^ copies/g, and a subsequent very slow further decrease or stabilization of *bla*_CTX-M-15_ copy numbers on lettuce or in soil, respectively. Four weeks after inoculation, still a high load of 6.3 × 10^2^ copies/g dry soil was detected, as well as an even higher load of 2.1 × 10^3^ copies/g lettuce leaf (dpi28, Figure 2B). In the control system, *bla*_CTX-M-15_ was detected in soil at 5.9 × 10^2^ copies/g on dpi0 and had almost doubled on dpi1 (9.2 × 10^2^ copies/g) to decrease again until dpi7 to 1.3 × 10^2^ copies/g. Thereafter, copy numbers stayed stable near the detection limit of 8.2 × 10^1^ copies/g until the last sampling, when the treated soil had reached similar levels (dpi28, Figure 2B). On control lettuce leaves, a low load of *bla*_CTX-M-15_ was detected initially (approx. 4 × 10^0^ copies/g) but was below the detection limit for lettuce in the subsequent weeks (< 1.8 × 10^−1^ copies/g).

**Manure.** Introduction of *E. coli* pEK499 via manure into the soil had a large impact on soil-borne *bla*_CTX-M-15_, increasing the soil’s background load from about 1.1 × 10^3^ copies/g to 1.4 × 10^7^ copies/g (Figure 2C). While *bla*_CTX-M-15_ copy numbers gradually decreased in treated soil until they reached 7.9 × 10^4^ copies/g on the day of lettuce planting, numbers in control soil fluctuated and were about the same at lettuce planting (9.4 × 10^2^ copies/g) as they were at the beginning of the quarantine period. Notably, despite the observed gradual decrease in *bla*_CTX-M-15_, treated soil still contained almost two orders of magnitude more *bla*_CTX-M-15_ than control soil on the day of planting. Copy numbers further developed in parallel for treated and control soil, both showing a slight decrease from the day of planting until dpi21. At dpi21, treated soil still contained significantly more *bla*_CTX-M-15_ copies than control soil (2.6 × 10^4^ vs. 9.5 × 10^1^ copies/g; Figure 2C). The high loads of *bla*_CTX-M-15_ in treated soil resulted in detectable *bla*_CTX-M-15_ in lettuce planted into this soil: gene copy numbers were stable during the first three days around 3.8 × 10^0^ copies/g and decreased to 1.0 × 10^0^ copies/g on dpi7 and below detection thereafter. In contrast, the lower load of *bla*_CTX-M-15_ contained in control soil did not lead to detectable *bla*_CTX-M-15_ in control lettuces at any time (< 1.8 × 10^−1^ copies/g; Figure 2C).

Overall, when introduced via water, *bla*_CTX-M-15_ persisted on lettuce for at least four weeks (study end), while untreated lettuce carried only initial low loads of *bla*_CTX-M-15_ that rapidly decreased below detection (Figure 2A,B). In soil, only high-level contaminated water resulted in significantly increased *bla*_CTX-M-15_ until dpi21. On the other hand, introduction via manure led to significantly increased *bla*_CTX-M-15_ levels in soil during the whole study period of 9 weeks but only initially significantly increased levels in treated compared to untreated lettuce (Figure 2C).

### 3.3. ARG Detection in Re-Isolated E. coli and Secondary Colonies

Representative colonies re-isolated from CrA(+) were screened for presence of *aadA5*, *bla*_CTX-M-15_, and *tetA* by triplex PCR. Colonies displaying *E. coli*-typical blue coloration on CrA(+) all yielded the expected triple band fingerprint, indicating presence of plasmid pEK499 (*n* = 133). Interestingly, apart from blue colonies, a number of white colonies grew on CrA(+), indicating resistance towards β-lactams and tetracycline. Representative white colonies also underwent triplex PCR, and 20 of 53 were positive for *aadA5*, *bla*_CTX-M-15_, and *tetA*. These isolates were therefore identified at species level by MALDI biotyping as potential recipients of plasmid pEK499, revealing that they all belonged to species *Morganella morganii*. Of note, nine of these strains originated from soil or lettuce of the control system, that is, with no contact to *E. coli* pEK499. *Morganella morganii* is known to carry chromosomal *bla*_CTX-M_ and *tetA* genes as well as various *aadA* genes located on plasmids or integrons [43].

## 4. Discussion

A lot is known about the prevalence and type of ARB and ARGs in various agricultural environments such as field soil, organic fertilizers, crops, or irrigation water [19,20,33,34,44,45]. A comprehensive analysis by Cerqueira et al. [46] describes the distribution of seven ARGs and *intI1* in soil, roots, leaves, and fruits/beans in an agricultural system, that is, the status quo without introducing an ARB in known quantities by using a specified contamination source. The authors could show a dilution effect from soil to fruits and beans of up to 1:10,000 but did not investigate the temporal dynamics during growth of the produce.

Further, many studies have described the occurrence of ARB and their resistance determinants in vegetables purchased from different markets and retailers, as nicely reviewed by Hölzel et al. [47]. However, studies recreating the chain of events at the interface between environment and plant and observing the temporal development of ARB and ARGs after their introduction are rare or investigated pathogenic *E. coli* O157:H7 (EHEC). We therefore investigated the time-dependent survival and persistence of the ESBL-producing *E. coli* pKE499 and its plasmid-borne *bla*_CTX-M-15_ in lettuce and soil, after introduction by irrigation or manure application.

### 4.1. Effect of Low- and High-Level Contamination by Irrigation

#### 4.1.1. Survival Dynamics of ESBL-*E. coli* pEK499

As expected, the level of contamination and amount of irrigation water used for inoculation strongly influenced the initial number of *E. coli* pEK499 recovered from soil and lettuce, with high-level contamination resulting in about 5 log CFU/g and 2 log CFU/g higher counts than low-level contamination in lettuce and soil, respectively (Figure 1, dpi0). Accordingly, *E. coli* pEK499 could be detected on lettuce longer after high- than after low-level contamination (until dpi7 as opposed to dpi0). However, surprisingly the bacterial numbers in soil decreased below the limit of detection (< 2.5 × 10^0^ CFU/g) for both treatments after dpi7, following a steep decline in the high-level contamination system. After dpi14, no culturable *E. coli* pEK499 were detected until the end of the experiment (dpi28).

Our high-level contamination is comparable to Bezanson and colleagues [48], who investigated survival of EHEC in romaine lettuce and soil after inoculation via irrigation. They used 100 mL of 10^6^ CFU/mL per lettuce head (vs. 10 mL per plant in this study), both of which resulted in similar initial levels on lettuce, probably due to significant runoff in the case of applying 100 mL per plant. In both studies, the decline on lettuce was steep leading to less than 10 CFU/g lettuce after 7 days, although numbers in Bezanson et al. varied between the two investigated field sites. In addition, in good accordance with our research, the decrease in soil found by Bezanson et al. was less steep than on lettuce, indicating as in our study that *E. coli* survives longer in soil.

In another study investigating EHEC survival on lettuce after plant inoculation via water, Solomon et al. [49] detected an effect of lettuce age at the time of inoculation—the older the lettuces, the higher the number of retained and surviving *E. coli*. When they inoculated at the same amounts as our low-level treatment and started with 30-day-old lettuces, they could detect *E. coli* by direct culturing until day 10. Although our lettuces were of comparable age at inoculation, we could detect *E. coli* by culturing only on dpi0 at very low levels (< 10^0^ CFU/g; Figure 1A). Solomon and colleagues did not determine bacterial counts on the day of inoculation. However, *E. coli* was detected at more than 10^3^ CFU/g on day 3. We therefore assume that their plants retained much higher *E. coli* loads than ours on their leaves during inoculation.

In yet another study testing EHEC, young or mature butterhead lettuces were inoculated by immersing or spraying, respectively [50], and growth chamber and greenhouse conditions were tested. They found that greenhouse conditions—which correspond approximately to the present study—were less conducive to *E. coli* survival than the more stable temperature and moisture in the growth chamber. They concluded that *E. coli* survival would be enhanced in greenhouses with sprinkler irrigation, where leaves are moisturized regularly. The absence of sprinkler irrigation in the present study might be one reason for the observed steep decrease in *E. coli* on the leaves.

#### 4.1.2. Persistence of bla_CTX-M-15_

As opposed to *E. coli* culturing, *bla*_CTX-M-15_ detection was much more sensitive. This resulted in (a) detection of background *bla*_CTX-M-15_ in control lettuce and soil and (b) detection until the end of the study (dpi28) on treated lettuce as well as control and treated soil (Figure 2A,B), which is three weeks after the last detection of culturable *E. coli* in any sample (Figure 1A,B). In the low-contamination experiment, treated soil was only slightly above background levels in the first three days, and *bla*_CTX-M-15_ levels were identical in the two types of soil from dpi7 and on, indicating that the introduced ESBL-producing *E. coli* did not affect the soil resistome in the long term. This is confirming the findings of Negreanu et al. [51], who concluded that treated wastewater did not alter the soil resistome significantly. The picture changed in the first phase when using the high-level inoculum, with treated soil exceeding background levels by about 2.5 orders of magnitude. However, *bla*_CTX-M-15_ in treated soil constantly decreased until it almost reached the level of control soil. This finding again confirms that, at least in the long term, the introduced ESBL-producing *E. coli* did not significantly alter the soil resistome. Contrasting our findings, a more comprehensive study by Dungan and colleagues [52] investigating six ARGs and *intI1* detected a significant enlargement of the ARG pool in soil treated with dairy wastewater. Unfortunately, the investigated ARG conferring β-lactam resistance (*bla*_CTX-M-1_) was not detected in the study at any time. It might well be that certain ARGs—in combination with other bacterial hosts—persist better in soil than the herein investigated *E. coli*-borne *bla*_CTX-M-15_.

The detection of *bla*_CTX-M-15_ on untreated young lettuce in both experiments most probably reflects an initial small contamination with soil during planting of the seedlings. This contamination, however, rapidly decreased below detection. On the other hand, *bla*_CTX-M-15_ clearly persisted on treated lettuce leaves and in both the low- and high-level contamination scenario. After an initial decrease, *bla*_CTX-M-15_ stayed virtually constant and clearly detectable until the end, despite long non-detectable *E. coli* pEK499 by culturing. Persistence of ESBL-producing *E. coli* on the leaves below detection alone cannot explain the high copy numbers of *bla*_CTX-M-15_, given the low copy number of pEK499 per cell. Alternatively, the ARG might have persisted in the absence of viable bacterial hosts. Finally, and most interestingly, *bla*_CTX-M-15_ might have spread and persisted in other hosts that are fitter for survival on leaves. In support of this assumption, the phyllosphere has been shown to be a hotspot for horizontal gene transfer [53]. We could not find another study showing persistence of *bla*_CTX-M-15_ on lettuce after introduction by irrigation water, as comparable studies seem to have focused on pathogenic bacteria such as EHEC.

### 4.2. Effect of Contaminated Manure Application

#### 4.2.1. Survival Dynamics of ESBL-*E. coli* pEK499

Culturable *E. coli* pEK499 was present in treated soil only. Interestingly, after a long log-linear decline numbers stabilized. Decline rates of *E. coli* in soil have been shown to depend mainly on environment (laboratory or field), soil type, and temperature [54]. Since the overall conditions remained stable throughout the experiment, the observed change in decline rate might reflect the existence of a certain carrying capacity for *E. coli* in the given system. Regarding the wait time between soil amendment and planting, Franz et al. [55] showed in a model for EHEC survival after introduction via manure that 60 days were needed for successful risk mitigation. In our system, 6 weeks were sufficient to avoid detection of *E. coli* pEK499 on the lettuce from the very first (Figure 1C, dpi0).

#### 4.2.2. Persistence of bla_CTX-M-15_

Manure application has been shown to influence the soil resistome significantly and in the long-term. In a field study, McKinney et al. [56] showed a significant increase in soil-borne ARGs after yearly dairy manure application. Nõlvak et al. who investigated fertilization of grassland soils concluded that cattle slurry and its digestate were considerable ARG sources, with ARGs decreasing after application but staying above background levels [57]. Finally, Chen and colleagues applied high-throughput sequencing and qPCR in a long-term field study and found that 130 ARGs were significantly increased after application of sewage sludge and chicken manure [58]. We could confirm these studies, as the introduced *bla*_CTX-M-15_ decreased but stayed above soil background levels, leaving treated soil with over two log higher copy numbers 9 weeks after amendment.

These increased copy numbers in soil were sufficient to result in detection of *bla*_CTX-M-15_ on lettuce during the first week after planting (Figure 2C). A field study by Fogler et al. showed a strong increase (up to 160-fold) in abundance of certain ARGs on lettuce and radishes grown in manure-amended soil [59] and concluded that proper composting was important. Further, Wang and coworkers detected ARGs in lettuce and endive harvested from manure-amended soil [60]. However, they did not analyze plants grown in non-amended soil making conclusions difficult, as the detected ARGs might have been present on plants irrespective of soil amendment. As shown, however, for enteric pathogens such as EHEC, internalization and migration of soil-borne ARB through the root system to edible plant portions can be assumed to take place [28], including ARB introduced via manure.

### 4.3. Good Agricultural Practice Recommendations

Current recommendations for good agricultural practices in handling surface water for irrigation or organic fertilizers for amendment vary worldwide. Concerning surface water used for irrigation, regular microbial testing is generally recommended, especially just before harvest [61,62,63,64]. When touching edible plant portions, irrigation before harvest should be avoided but the recommended wait time varies from a few hours to one week [61,63]. Our findings show a rapid decline of water-introduced *E. coli* on the lettuce, suggesting that a short period of 24 h would suffice in case of 10^4^ CFU/mL of water, whereas up to one week is required in case of 10^6^ CFU/mL (Figure 1). Further, the introduced *bla*_CTX-M-15_ persisted for at least four weeks on the lettuce. Of note, water containing 10^4^ *E. coli* per mL is by some guidelines already considered above the threshold to be used for irrigation with direct leaf contact [62], emphasizing the importance of avoiding leaf contact whenever possible.

For soil amendment using raw manure, when not discouraged, incorporation into the soil is recommended and a waiting period between application and harvest must be observed. These periods range from 6 weeks to several months [23,24]. In our study, culturable *E. coli* were detectable for at least 9 weeks after manure application, and so was the monitored ARG *bla*_CTX-M-15_. These findings are in support of long waiting periods of at least two months. As numbers of culturable *E. coli* stabilized after five weeks (Figure 1C, day -7) and did not drop below detection until dpi21, an observation time longer than 9 weeks is needed to determine how long viable *E. coli* can persist in the soil after their introduction via manure.

## 5. Conclusions

The present study demonstrated a long-term persistence of plasmid-borne, ESBL-encoding gene *bla*_CTX-M-15_ in soil and lettuce after its introduction via irrigation water (≥ 4 weeks) or manure (≥ 9 weeks). When introduced via manure into soil, the plasmid-carrying, culturable *E. coli* persisted at low counts in soil enabling a re-contamination of lettuce at harvest, but more importantly, introduction via overhead irrigation led to persistence of *bla*_CTX-M-15_ for at least four weeks on the edible lettuce portions, despite the long absence of culturable ESBL-producing *E. coli*. These findings underline the importance of monitoring ARB and their corresponding resistance genes in irrigation water and organic fertilizers to avoid their introduction, persistence, and possible spread of resistance determinants until the harvest of raw-consumed fresh produce.

## Figures and Tables

**Figure A1:**
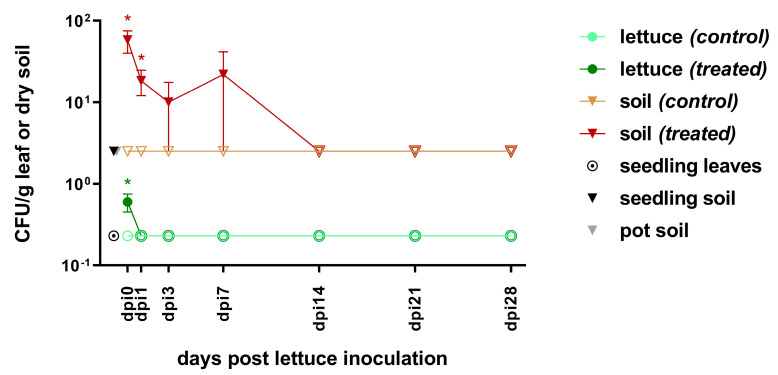
Detailed view on the survival of *E. coli* pEK499 in soil (-▼-) and lettuce (-●-) from its introduction until harvest, after low-level contamination via irrigation water. Empty symbols indicate values below the respective limit of detection. Error bars indicate standard error of the mean (SEM, *n* = 3). Significant differences between treatment and control (*p* < 0.05) are marked with an asterisk.

## Appendix A

Figure A1: Detail view on survival of *E. coli* pEK499 in soil and lettuce after low-level contamination via irrigation water, Table A1: Primers for triplex PCR, Table A2: Cycling conditions for triplex PCR.

**Table A1:** Primers for triplex PCR detection of *aadA5*, *bla*_CTX-M-15_, and *tetA* present on plasmid pEK499 and amplification of 16S rRNA gene.

Primer	Sequence (5’-3’)	Product Size (bp)	Origin
aadA5_F	CTG CGA CAC TGG ACA CAA TC	502	This study
aadA5_R	CGA GCA AGA GCA AGA ACG AC		
CTX-M-15_F	CCT TAG GTT GAG GCT GGG TG	760	This study
CTX-M-15_R	TTG TTA GGA AGT GTG CCG CT		
tetA_F	CAG GCA GAG CAA GTA GAG GG	634	This study
tetA_R	CCT CAA TTT CCT GAC GGG CT		
27F	AGA GTT TGA TCM TGG CTC AG	1505	[65]
1492R	CGG TTA CCT TGT TAC GAC TT		

**Table A2:** Cycling conditions for triplex PCR detection of *aadA5*, *bla*_CTX-M-15_, and *tetA* present on plasmid pEK499.

98 °C	Initial Denaturation	30 sec	
98 °C	Denaturation	10 sec	30 cycles
65 °C	Annealing	30 sec	
72 °C	Extension	30 sec	
72 °C	Final Extension	10 min	
